# A systematic analysis of genomics-based modeling approaches for prediction of drug response to cytotoxic chemotherapies

**DOI:** 10.1186/s12920-019-0519-2

**Published:** 2019-06-17

**Authors:** Joshua D. Mannheimer, Dawn L. Duval, Ashok Prasad, Daniel L. Gustafson

**Affiliations:** 1 School of Biomedical Engineering, Colorado State University, Fort Collins, 80523 CO USA; 2 Flint Animal Cancer Center, Colorado State University, Fort Collins, 80523 CO USA; 3 Department of Chemical and Biological Engineering, Colorado State University, Fort Collins, 80523 CO USA; 4 Department of Clinical Sciences, Colorado State University, Fort Collins, 80523 CO USA; 5 University of Colorado Cancer Center Developmental Therapeutics Program, University of Colorado, Aurora, 80045 CO USA

**Keywords:** Cytotoxic chemotherapies, Machine learning, Genomic modeling, Drug response, Cancer

## Abstract

**Background:**

The availability and generation of large amounts of genomic data has led to the development of a new paradigm in cancer treatment emphasizing a precision approach at the molecular and genomic level. Statistical modeling techniques aimed at leveraging broad scale in vitro*,* in vivo*,* and clinical data for precision drug treatment has become an active area of research. As a rapidly developing discipline at the crossroads of medicine, computer science, and mathematics, techniques ranging from accepted to those on the cutting edge of artificial intelligence have been utilized. Given the diversity and complexity of these techniques a systematic understanding of fundamental modeling principles is essential to contextualize influential factors to better understand results and develop new approaches.

**Methods:**

Using data available from the Genomics of Drug Sensitivity in Cancer (GDSC) and the NCI60 we explore principle components regression, linear and non-linear support vector regression, and artificial neural networks in combination with different implementations of correlation based feature selection (CBF) on the prediction of drug response for several cytotoxic chemotherapeutic agents.

**Results:**

Our results indicate that the regression method and features used have marginal effects on Spearman correlation between the predicted and measured values as well as prediction error. Detailed analysis of these results reveal that the bulk relationship between tissue of origin and drug response is a major driving factor in model performance.

**Conclusion:**

These results display one of the challenges in building predictive models for drug response in pan-cancer models. Mainly, that bulk genotypic traits where the signal to noise ratio is high is the dominant behavior captured in these models. This suggests that improved techniques of feature selection that can discriminate individual cell response from histotype response will yield more successful pan-cancer models.

**Electronic supplementary material:**

The online version of this article (10.1186/s12920-019-0519-2) contains supplementary material, which is available to authorized users.

## Background

The introduction of cDNA microarrays launched a new era of genomic studies in biological systems [1, 2]. This revolutionary new technology allowed researchers to collect vast amounts of data to characterize the genomic landscape fundamental to biological processes. The power of this technology was soon realized to have broad implications in the study of cancer providing insight into the genomic nature of the disease [3–5]. Over the past few decades there has been a concerted community effort to collect both in vivo *and* in vitro data characterizing the molecular blueprints for a variety of cancers [6, 7]. This work has spawned countless new insights and has paved the way for a new paradigm of cancer treatment involving precision approaches [8].

The term “Big Data” refers to the collection and storage of large amounts of information for analysis providing insight for a variety of applications [9]. The mathematical, statistical, and computational techniques to analyze and extract this information from large sets of complex data encompasses the field of statistical learning having application in science, business, and technology [10]. The ability of statistical learning theory to find useful information in large, complex, and often noisy datasets make it a popular biomedical research area with clear clinical applications [11] including several in cancer diagnostics and treatment [9, 12, 13]. A specific area of research has focused on the utilization of statistical learning to predict successful treatment options based on patient and disease specific clinical biomarkers [5, 14–16].

High throughput technologies have allowed researchers to profile the genomics of tumor-derived cell lines and test chemosensitivity to a variety of anti-cancer agents in vitro, most notably the National Cancer Institute 60 (NCI60) and the Genomics of Drug Sensitivity in Cancer (GDSC) cell line panels. Several studies have indicated the ability of in vitro data to predict patient response in multiple cancers [17–20]. Therefore, in vitro drug response data offers a simplified format to uncover clinically relevant cancer drug relationships. Thus, models that can accurately capture behavior of in vitro experiments are essential to elucidate genomic signatures that can be further applied in more complex clinical models.

To date, one of the most comprehensive analysis of computational methods for predicting drug response with in vitro data was a community based challenge sponsored by the Dialogue on Reverse Engineering Assessment and Methods (DREAM) and National Cancer Institutes (NCI) (referred to as the DREAM-NCI challenge) [21]. This challenge tasked 44 different research teams to build and train a predictive algorithm given gene expression, DNA methylation, mutation, copy number, protein abundance, and drug response for 35 breast cancer cell lines for 28 different known anti-cancer agents. The methods were then assessed on their ability to predict drug response for the 28 agents on 18 independent breast cancer cell lines. The resulting models highlighted some of the most advanced and cutting edge statistical learning techniques with the best model using Bayesian multi-task multiple kernel learning (MKL). However, the third best model differed in performance by only 2.3% using only weighted Pearson correlation between feature sets with drug response to make predictions. Overall, the DREAM-NCI challenge demonstrated the ability of statistical learning techniques to capture and predict drug response in in vitro environments.

The DREAM-NCI challenge illustrates the balancing act between complexity and simplicity that often presents itself in computational modeling. As “Big Data” takes off, more complex computational techniques will be developed offering new opportunities in precision oncology. However, to fully utilize and develop these techniques a firm understanding of how basic modeling principles influence performance is essential. Biological processes consist of complex dynamic interactions in a high dimensional system. Non-linear methods have the ability to capture complex interactions between players, however, in high dimensional systems these methods have a tendency to incorporate noise leading to over-fitting. Alternatively, linear methods are more robust to over-fitting but at the cost of potentially missing important non-linear interactions. Furthermore, the high dimensional nature of biological data sets presents challenges in the ability to pinpoint covariates that are most informative to the underlying processes being modeled.

Insights into the molecular nature of cancer has driven a precision approach to cancer pharmacology by capitalizing on specific driver mutations exhibited by certain cancers [22–24]. This strategy had been successful in a number of specific instances and continues to be an active area of research and drug development [25, 26]. Cytotoxic chemotherapies were some of the earliest drugs developed for the treatment of cancer and continue to play an important role in cancer therapy [27–30]. However, the success of these drugs, as with all therapies, still varies [31]. The toxicity associated with these drugs produce substantial side effects and can diminish quality of life for many patients; thus, a precision approach that can identify patients who would benefit could greatly improve the quality and efficacy of treatment. In vitro drug assays have become a standard approach to identifying compounds with potential therapeutic benefit [17, 18, 20]. Opposed to targeted agents, mutations are poor predictors of efficacy for cytotoxic agents [32] and genomic signatures have proven to show promise as predictors in cytotoxic agents [33, 34]. Therefore, genomic data driven models that can accurately predict chemosensitivity to in vitro cell line assays of cytotoxic agents serve as a foundation for improving predictive models in patients.

Here we describe a systematic, pragmatic approach to identify the key components driving model performance when using genomic profiles to predict drug response in cytotoxic agents. While statistical learning offers a vast amount of possible techniques we simplify the approach by breaking down models into two fundamental aspects; the trade off between linear and non-linear modeling techniques and the influence of feature selection via filter based selection methods. While, our approach is by no means an exhaustive survey of all possible techniques and approaches, our studies illustrate how simple approaches to modeling can offer valuable insight. Mainly we demonstrate that for a given population of cells the association between histotype and drug response is indicative of model performance. The dominance of these traits have important implications when assessing model performance and may prove instructive in the development of new techniques for modeling drug response across multiple cancers.

## Methods

### Preprocessing

The Genomics for Drug Sensitivity in Cancer (GDSC) is comprised of over a 1000 cancer cell lines with response data to 138 anticancer drugs. The available CEL files containing gene array data using Affymetrix Human Genome U219 array were downloaded at [35]. Using the “affy” R package the CEL files were normalized using Robust Multi-Array Average (RMA) algorithm [36]. The data was further corrected for batch variability using COMBAT of the “sva” R package [37]. Cells that occurred in duplicate were averaged resulting in a final gene expression matrix with 968 cell lines and 49,386 genomic features. Likewise, for the NCI60, CEL files containing gene array data from Affymetrix Human Genome U133 2-plus array were downloaded from the CellMiner database [38, 39]. A total of three CEL files were available for each NCI60 cell line, again the data was normalized using RMA [36] and batch corrected using COMBAT [37] the resulting data was then averaged over the three replicates to give a final gene expression value for each gene and cell. For our analysis in the GDSC we chose 15 cytotoxic chemotherapies Table 1. The IC50 data was downloaded from [35]. The NCI60 has 61 FDA approved cytotoxic agents [40], the drug response data again downloaded from CellMiner. For the majority of drugs multiple IC50 measurements were made on multiple cell lines so the final IC50 represents an average over all measurements. For several of the drugs a significant number of the cell lines had the same reported IC50 leading to minimal variability and as such these drugs were discarded. This left a total of 39 drugs, a list of which can be found in Additional files 1, and 14 were also had data in the GDSC. Several cell lines in multiple drugs in the GDSC reported IC_50_ above the maximum concentration experimentally tested and were not included in any of the models. Given the final number of cell lines as reported in Table 1. 75% of cell lines were randomly chosen and assigned to the training/validation set and the remaining 25% were assigned to the testing set. This was performed six times generating six non-overlapping test-train/validation splits. Likewise, in the NCI60 six random training/validation sets consisting of 75% of the data with the remaining 25% left out for testing. To ensure the presence of each histotype in both testing and training sets, 75% of each histotype was reserved for training and validation with the remaining 25% in the test set. Prostate cell lines were removed because measurements were limited. Both in the GDSC and NCI60 these generated datasets were used on all models within a given drug.

**Table 1:** Cytotoxic Drugs and number of cell lines

Drug	Abbreviation	Number of Cell Lines
Bleomycin	BLM	632
Bortezomib	BTZ	331
Cisplatin	CIS	146
Cytarabine	CYT	515
Docetaxel	DTX	555
Doxorubicin	DOX	738
Etoposide	ETP	643
Gemcitabine	GEM	583
Methotrexate	MTX	216
Mitomycin C	MMC	759
Paclitaxel	PTX	227
Vinblastine	VBL	719
Vorinostat	VOR	728
SN-38	SN-38	698
5-Fluorouracil	5-FU	409

15 Cytotoxic agents and the number of cell lines with experimentally determined IC50’s for each drug. Training set comprises 75% of the total data while the testing data accounts for the remaining 25%

The choice to limit our analysis to cytotoxic chemotherapies was three-fold; first, as opposed to molecularly targeted therapies, cytotoxic chemotherapies work broadly to inhibit cell proliferation and the mechanisms of action are not dependent on specific driver mutations [22, 23]. This has been demonstrated in the NCI60 where mutation status was shown to be a poor predictor of drug response in cytotoxic chemotherapies [32]. Second, a study in “The Cancer Genome Atlas” concluded that “the information content content from copy number aberrations, miRNA’s and methylation is captured at the level of gene expression and protein function” [41]. Lastly, several analyses have suggested that gene expression data accounted for the majority of variability in predictive model outcomes [21, 42]. By restricting the study to cytotoxic agents complications that arise from data redundancy could be minimized while also eliminating challenges in integrating different data types. Thus, variability in model performance could directly be attributed to methodological experimental factors.

### Model construction

**Fig. 1:**
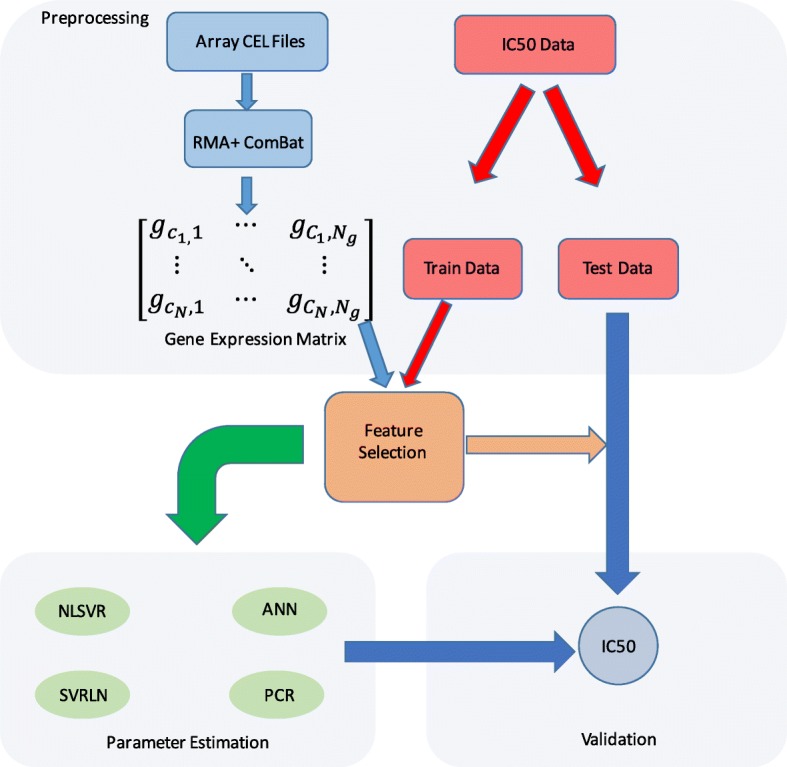
General workflow: The general workflow used to build models

Figure 1 outlines the basic procedure used to build all models. Feature selection was performed on the training data followed by model training after which the model was validated using the independent test set. Four different regression methods were used for model development including two linear methods, Principle Components Regression (PCR) and Support Vector Regression with a linear kernel function, and two non-linear methods including non-linear Support Vector Regression (NLSVR) and Artificial Neural Networks (ANN). We implemented 3 different feature selection strategies with all four algorithms and an additional seven on our best performing linear model (PCR) and non-linear model (NLSVR). These feature selection methods are summarized in Table 2 Feature selection was performed in python 2.7 and a generic python 2.7 script was used to read, organize, and write the model output. PCR was implemented in R version 3.2.4 using the PLS package with the number of components chosen by 10-fold Monte Carlo cross validation. Both NLSVR and SVRLN were implemented with scikit-learn version 18.1 [43]. For NLSVR parameter optimization was performed on three separate parameters amounting to 210 different three parameter combinations using 10-fold Monte Carlo cross validation. Likewise, SVRLN was optimized over two parameters for 30 different combinations using 10-fold Monte Carlo cross validation. A single layer ANN with 20 hidden nodes was implemented using the Keras package in python. To combat over-fitting dropout was implemented using 10-fold Monte Carlo cross validation with dropout rates 0,10,25, and 50% of total nodes chosen by 10-fold Monte Carlo cross validation. All parameter optimization and model training was performed using only the training data and the independent testing data was used to assess model performance.

**Table 2:** Feature selection methods

Selection Method	Description
No feature selection (NO FS)	All probes used with a total of 49,386 probes.
Differentially Expressed genes (DEGs)	Array probes that have a statistically significant Spearman correlation *P* < 0.05 with drug response
LIMMA	Linear Empirical Bayes with a modified t-statistic as implemented in the LIMMA Bioconductor package in R. Genes were selected by running LIMMA on the top and bottom 25% sensitive and resistance cell lines. A false discovery rate of 5% was chosen as a cutoff.
Bonferroni Correction (BC)	Bonferroni Correction ${\rho}_{BC}=\frac{\alpha }{m}$ where *α* is significance level of 0.05 and *m* is the number of features tested, 49,386. *ρ*_*BC*_ = 1.0 *x* 10^−6^
DEG Bootstrap (BS)	Array probes which have a statistically significant Spearman correlation P < 0.05 in fifty random subsets containing 75% of the training data
Histotype specific Bootstrap (BS-Hist)	50 subsets of the training data were generated such that each subset contained only one cell from a specific histotype. Probes that have a significant Spearman correlation P < 0.05 in 50% of the splits were selected. ** Data not shown, reported Additional file 2
Maximum Relevance Minimum Redundancy (MRMR)	Maximum Relevance Minimum Redundancy. 1000 Probes are chosen such that they have a maximum correlation with drug response with minimal cross-correlation with other chosen probes.
Control 1 (CTR1)	Probes are randomly selected from all 49,836 probes equal to the number of DEGs for each model/trial. For example, bleomycin dataset 1 yielded 5377 DEGs in DEG feature selection thus 5377 probes are selected randomly in control 1 experiments.
Control 2 (CTR2)	The compliment of DEGs. For example, for bleomycin dataset 1 control 2 genes would include 38,009 probes excluded form the 5377 probes selected as DEGs.
Random Control (RCTR)	A number, N, of probes equal to the number of DEGs are randomly selected. This gives N vectors with each entry corresponding to a cell line in the training set. This vector is then shuffled randomly such that the original value is no longer associated with the same cell yielding a feature matrix that is arbitrary.
Histotype Only (HIST)	Each cell line is associated with a 55 dimensional vector where the nth entry is 1 if the cell comes from the corresponding histotype and 0 otherwise. (One hot encoded)

A summary and definition of the different feature selection methods discussed in the results section. The abbreviations that will be used in the text to refer to these methods are in prentices

Gene expression data is inherently high dimensional, presumably, a given biological response, such as drug response, is influenced by a subset of the total genes. Feature selection provides a means to reduce the number of covariates systematically favoring features that are most relevant to the problem. This often leads to more favorable outcomes by eliminating features that only contribute to noise leading to a more robust signal and a decrease in over-fitting. Filter based feature selection attempt to associate a given feature (gene) to a targeted output (drug response) based on statistical inference. Many such of these algorithms exist for gene expression data [44] and contextually amount to looking at two or more populations (i.e. drug resistant, drug sensitive cells) and determining if a given feature is statistically different between groups. Such methods are often applied to classification problems but can be generalized to continuous responses by looking at populations with distinct responses. However, this method requires reformatting the problem into a binary classification problem and assuming it can be generalized to a continuous response. Alternatively, correlation based feature selection methods (CBF) are more aptly suited to continuous processes by looking at the statistical relationship between a covariate and target variable based on correlation [45].

To asses the affects of reducing features in our models we use several CBF feature selection methods. First we implemented the non-parametric Spearman correlation using a cutoff of *p* < 0.05 to determine as set of differentially expressed probes (DEGs) using the statistics package in scipy 0.17.0. We compare this to a standard method of isolating probes with distinct difference between the 25% of cells with the greatest IC50s (resistant) and the lowest IC50 (sensitive) using the R Limma package [46] with a false discovery rate q = 0.05. In order to assess the influence of feature selection we performed three control experiments. For the first control (CTR1) we randomly selected a number of probes that corresponded to the the same number of DEGs for a given experiment. The second control (CTR2) consists of all probes that are not selected as DEGs. Lastly, we perform a random control (RCTR) by shuffling the gene array matrix leaving the response vector untouched and then random selecting the same number of probes used in DEGs and CTR1. We address multiple testing by using a Bonferroni correction for p cutoff in the spearman correlation. Additionally, we explore a bootstrapping method to decrease false discovery rate (FDR). Lastly we apply a maximum relevance minimum redundancy (MRMR) algorithm [47]. All feature selection methods were applied to the training set prior to model fitting. A summary of the different feature selection methods as summarized in Table 2.

### Analysis

**Fig. 2:**
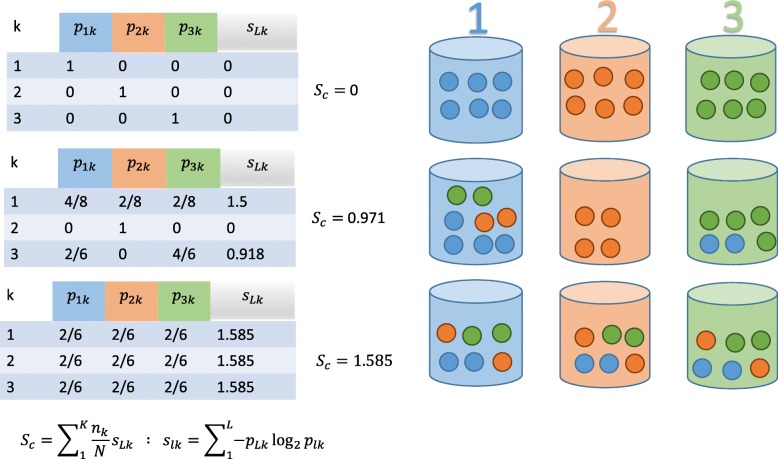
Cluster Entropy: Illustration of how cluster entropy, S_c_, is calculated. It is a measure of cluster homogeneity, in this case, how many cells of the same histotype are placed in the same cluster

The performance of each model was assessed using the Spearman correlation coefficient between the predicted and measured IC50 values in the testing set using the scipy statistics package version 0.17.0, *p* values were calculated within the statistics package using a student’s t distribution. Additionally, we also calculated a Mean Absolute Difference metric (MAD). The MAD scores were generally reflective of the Spearman correlation, therefore, we have chosen to report the Spearman correlation, as it better highlights particular patterns in the data in the main paper but MAD values for all models can be found in the Additional file 2 K-means clustering was performed using the clustering package in scikit-learn [43]. The ability of a given set of genes to assign cells of the same histotype to the same cluster was determined using Clustering Entropy, *S*_*c*_ [48], which is defined and conceptually illustrated in Fig. 2 *S*_*c*_ has a minimum value of 0 when histotypes are perfectly clustered together. A theoretical maximum *S*_*c*_ occurs when each cluster contains a uniform distribution of samples from different histotypes, however, since samples are not uniformly distributed across histotypes and each dataset contains a different distribution of histotypes the maximum value was estimated using the random control for each dataset and the values reported are normalized consisting of the *S*_*c*_ of the given dataset divided by the *S*_*c*_ of the random control. Note that by this definition the normalized *S*_*c*_ can be greater than one.

## Results

### Regression models

**Fig. 3:**
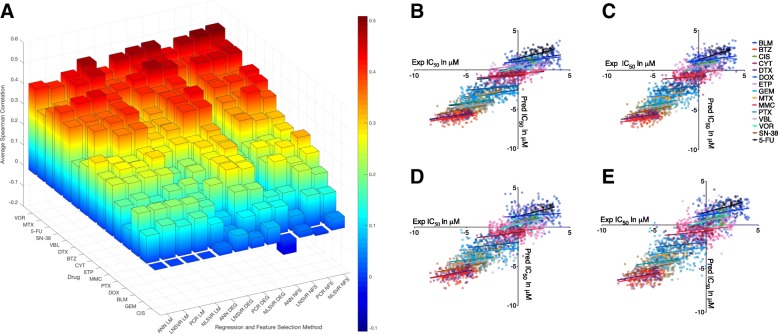
Model performance by method and Drug: **a** Average spearman correlation coefficients for four different regression methods over three different methods of feature selection. **b**-**e** Predicted versus Measured IC50 values for each of the fifteen drugs using DEG genes. **b** NLSVR, **c** PCR, **d** SVRLN, **e** ANN

Individual Spearman correlations between measured and predicted IC50 values ranged from 0.64 to − 0.345 with 51–84% percent of the models showing significance (*P* < 0.05). While NLSVR (0.316–0.331) yielded higher average Spearman correlations than PCR (0.297–0.316) and SVRLN (0.27–0.285), the difference on a per drug basis was minimal (Fig. 3a). ANN showed significant drops in performance (0.144–0.266) compared to the other three methods especially when no feature selection was performed, while, the gap narrowed upon the introduction of feature selection, performance was still substantially less, most notably when compared with NLSVR and PCR (Fig. 3a, Table 3).

**Table 3:** Model Performance

	NLSVR			PCR			LNSVR			ANN		
	NFS	DEG	LIM	NFS	DEG	LIM	NFS	DEG	LIM	NFS	DEG	LIM
BLM	.207	.202	.202	.239	.208	.208	.151	.1	.209	.147	.17	.21
BTZ	.38	.404	.365	.422	.399	.354	.332	.326	.232	−.009	.299	.24
CIS	.05	.08	N/A	−.009	.047	N/A	.03	.079	N/A	−.066	.034	N/A
CYT	.313	.32	.279	.32	.281	.256	.337	.291	.269	.226	.266	.291
DTX	.422	.44	.408	.367	.409	.382	.357	.319	.359	.185	.318	.207
DOX	.273	.27	.117	.243	.285	.106	.27	.226	.103	.115	.173	.096
ETP	.289	.302	.294	.248	.291	.263	.238	.219	.273	.209	.195	.246
GEM	.143	.139	.166	.153	.117	.143	.07	.063	.165	.131	.119	.134
MTX	.461	.455	.462	.431	.435	.433	.417	.388	.338	.411	.391	.322
MMC	.237	.302	.244	.264	.269	.25	.27	.224	.239	.203	.153	.248
PTX	.32	.27	.198	.287	.282	.159	.233	.170	.191	−.106	.211	.177
VBL	.44	.403	.399	.408	.398	.37	.398	.339	.371	.112	.302	.363
VOR	.509	.495	.486	.5	.487	.439	.484	.471	.404	.445	.42	.42
SN-38	.383	.417	.409	.379	.391	.443	.397	.404	.429	.01	.327	.402
5-FU	.463	.464	.40	.455	.484	.354	.451	.438	.337	.309	.409	.365
AVG	.326	.331	.316	.314	.319	.297	.285	.27	.28	.144	.252	0.266

Average spearman correlations across six different testing sets for all regression and feature selection methods. This data is graphically displayed in Fig. 3

Correlation based feature selection (P < 0.05) decreased the number of features by an average of 77% (range 39 to 96%) with the fewest features for cisplatin and most for vorinostat Table 4 Model performance was increased for ANN (63%) increasing the average Spearman correlation by 63% with only a modest increase for NLSVR (1.5%) and PCR (1.6%). The decrease in features had a minor negative impact on SVRLN (9%) performance. Feature selection by use of the R package Limma was substantially more restrictive than the DEG criteria, leading to an 99% decrease feature number, yielding no features for cisplatin. Despite this substantial decrease in genes, only a 9% average decrease in correlation was observed with similar effects to NLSVR, PCR, and SVRLN (~ 11%) and minimal effects to ANN (1.6%) in comparison to the top performing feature selection method.

**Table 4:** Feature Selection and number of features

Drug	DEG	Limma	BC	BS	BS Hist
BLM	5216.5; 4414; 6314	18.2; 11; 32	2.5; 0; 6	178.3; 117; 298	8.7; 6; 15
BTZ	11522.2; 10087; 12191	233.3; 124; 414	93.3; 52; 132	1626.5; 1208; 1888	442.5; 820; 144
CIS	2354.8; 1835; 2890	NA	NA	20.5; 11; 32	188.8; 103; 329
CYT	12954; 8675; 16,844	371.8; 123; 826	174.5; 12; 414	2063.1; 714; 3704	571.8; 104; 1374
DTX	15292; 13054; 16938	330.5; 127; 549	398.8; 147; 625	2979.8; 1958; 3718	794.7; 585; 955
DOX	4983.3; 4469; 5368	12.8; 8; 18	2.5; 0; 6	167.8; 145; 201	27.5; 50; 13
ETP	11271.5; 10707; 12744	178.7; 91; 284	45.8; 32; 63	1254; 1024; 1720	146.8; 22; 269
GEM	5728.7; 4099; 7423	7; 2; 17	3.2; 1; 8	185.2; 82; 342	34.2; 5; 79
MTX	15727.3; 13692; 18906	130; 15; 203	398.8; 11; 687	3153.5; 2010; 4378	2329.3; 2748; 1372
MMC	6515.8; 4936; 8485	30.8; 8; 95	6.7;3; 14	332.2; 127; 629	26.5; 5; 116
PTX	6175.2; 4997; 7344	12; 4; 20	4.7; 1; 8	295; 465; 162	293; 136; 392
VBL	15207.7; 12488; 17196	201.8; 114; 284	447.33; 124; 716	3094.8; 1830; 4142	510; 231; 809
VOR	29935.2; 29461; 30337	5796.7; 4971; 6284	8165.2; 7274; 8690	15931.3; 14934; 16,448	4107; 3213; 5604
SN-38	13501.5; 11919; 16510	190.8; 108; 424	315.3; 153; 738	2577.2; 1761; 4286	193.5; 28; 149
5-FU	16218.8; 14888; 17706	236.5; 142; 320	333; 208; 480	3240.8; 2595; 3928	773.7; 550; 1084

The number of by feature selection method. Each cell contains the mean, maximum, and minimum number of features (Mean; Minimum; Maximum)

### Feature selection and model performance

**Fig. 4:**
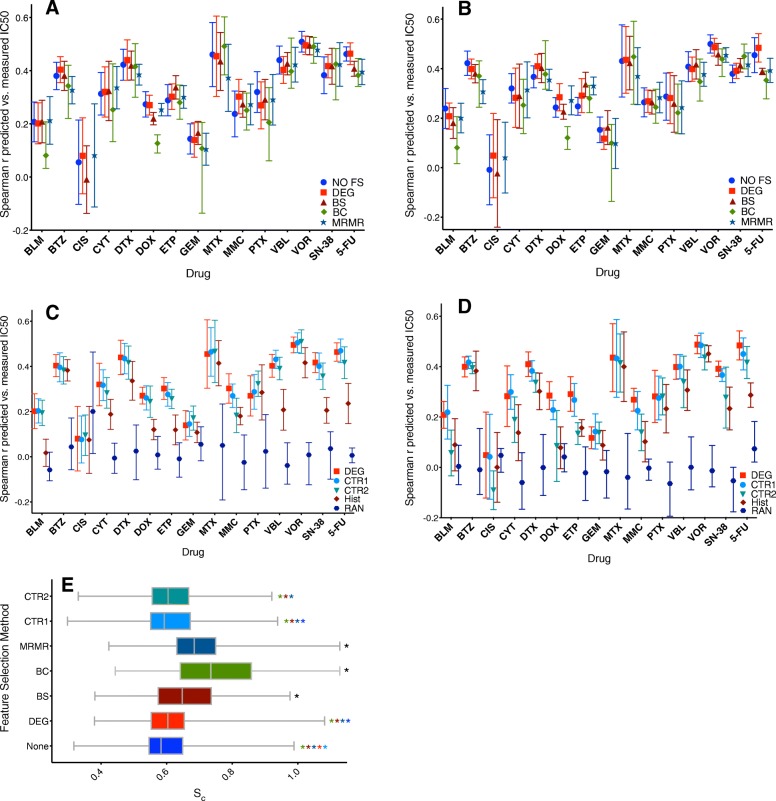
Feature selection methods and controls: **a**-**b** Spearman Correlation Coefficients for different feature selection methods NLSVR (**a**), PCR (**b**). **c**-**d** Spearman Correlation Coefficients for control models NLSVR (**c**), PCR (**d**) the placement of the symbol indicates the mean with the ends representing the range. **e** Cluster Entropy (S_c_), indicative of how well cell lines of the same histotype cluster using k-means. Comparable S_c_ as well as little difference in r indicate that histotype recognition drives model performance. S_c_ is relative to the random control (RCTR) where S_c_ = 1, perfect clustering would have a S_c=_0. The asterisks indicate significance (*p* < 0.05) between the method and alternative models, indicated by color (black indicates is was significant compared to all other methods), using a non parametric Wilcoxon match-paired rank test. The calculated *p* values can be found in Additional file 3

The results from BC, BS, and MRMR models for NLSVR and PCR in (Fig. 4a and b). Compared to DEG and NO FS models, all three methods yielded lower average Spearman correlations. BC criteria reduced features by and average of 98.6% with no selected features for cisplatin datasets as well as two datasets for bleomycin and a single dataset for doxorubicin (Table 4). The use of BC selected features decreased the overall average Spearman correlation by 11.4% (0.3513 to 0.3112) for NLSVR and 12.2% (0.3406 to 0.2991) across identical datasets using DEG selected features. The most dramatic decreases in performance was seen in bleomycin, cytarabine, doxorubicin, and 5-Fluorouracil (Fig. 4a and b). A small increase in performance was seen for methotrexate in NLSVR models. Despite the decreased performance 80% of the models had significant correlations (*P* < 0.05) between experimental and predicted IC50 values.

Bootstrap methods resulted in an average 95% decrease of features. Performance decrease was slightly less than that of BC selected genes with an average decrease of in NLSVR (3.4%) and PCR (4.4%) models. A substantial decrease in performance was observed for cisplatin while an increase in performance was seen in etoposide, gemcitabine, and paclitaxel in comparison with DEG models for both NLSVR and PCR (Fig. 4). Likewise, the modified MRMR algorithm was used to select 1000 features, representing a 98% decrease in features. The drop in performance was similar to that seen with both BC and BS for NLSVR (6.3%) and PCR (6.9%). The general decrease in performance correlated directly with the reduction in the number of genes; however, even a maximum 98.6% decrease in features only resulted in 11.4% drop in performance for NLSVR and 12.2% for PCR. Additional methods of feature selection that attempted to take the histotype into account yielded similar performances (Additional file 2).

### Influence of CBF feature selection

In order to gain insight into the overall influence of correlation based features we tested several sets of control features on the datasets, designed to address the following questions. First, what was the benefit of using DEGs compared to the same number of randomly selected genes (CTR1)? Second, how influential was the inclusion of correlation based features, thus, what would be the effect of using those genes that had no significant relationship to drug response (CTR2)? Lastly, were these relationships simply an artifact that was introduced during the collection, preprocessing, and normalization of the data, and thus what happens if all causal relationships are removed (Random Control)?

The use of CTR1 genes resulted in a decreased performance < 1% (0.331 to 0.329) for NLSVR and a 3.1% (0.319 to 0.309) for PCR in average Spearman correlation. With respect to DEGs in NLSVR, CTR1 genes led to comparable average Spearman correlations for each drug and exceeded DEGs in certain drugs such as methotrexate, paclitaxel, vinblastine, and vorinostat (Fig. 4c.). Likewise, for PCR, small increases in average Spearman correlation was seen for bleomycin, bortezomib, cytarabine, and gemcitabine while a minimal decrease for other drugs (Fig. 4d.). Surprisingly, removing features with a-priori significant statistical relationships with drug response had little overall negative effects on the average performance of NLSVR models (4.8%) with cisplatin, gemcitabine, paclitaxel, and vorinostat yielding better average performances than the same DEG models (Fig. 4c.). However, the performance of CTR2 models in PCR dropped significantly by 26% (0.319 to 0.236) compared to DEG models, however, 64% of the models had significant correlations. Nonetheless, comparable performances were observed in several drugs including bortezomib, docetaxel, methotrexate, vorinostat, 5-fluorouracil, and gemcitabine while other drugs such as bleomycin, docetaxel, cisplatin, and SN-38 saw dramatic decreases in performance (Fig. 4d.). Lastly, by randomly assigning expression values to cell-lines (Random Control), there was a significant loss in the predictive ability of the model with average Spearman correlations of 0.0185 for NLSVR and − 0.007 for PCR (Fig. 4c and d.). The loss in predictive capability when the gene-cell line relationship is removed demonstrates that our models are clearly capturing a genomic signature that is indicative of drug response.

### Histotype is linked to drug response

**Fig. 5:**
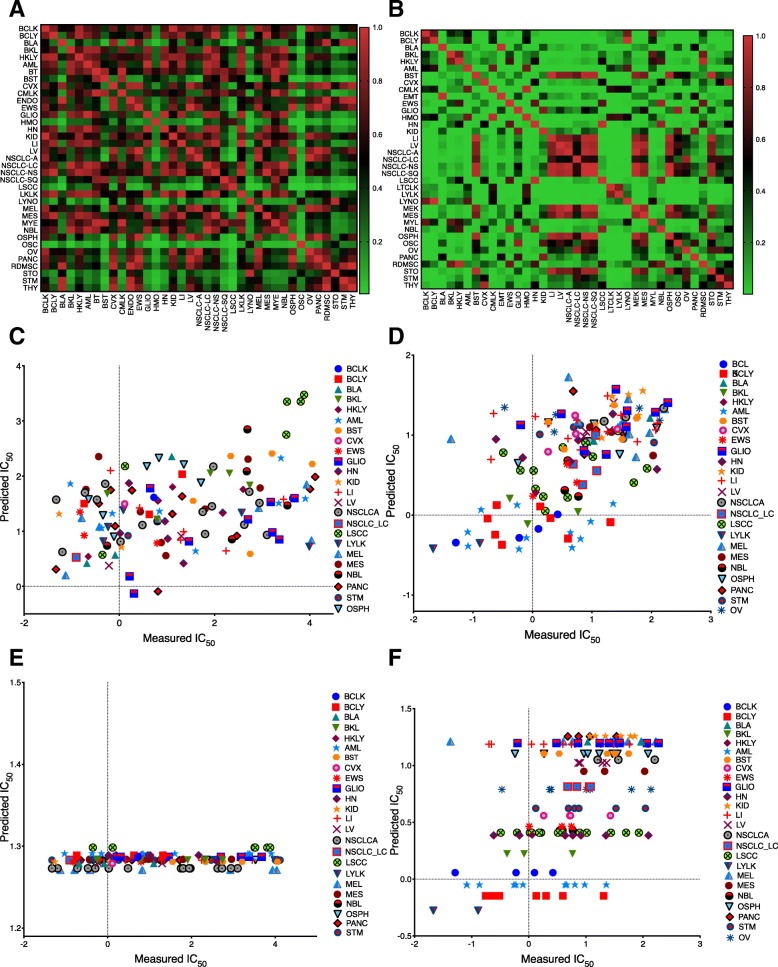
Histotype influence on drug response. **a**-**b** *P* values for pairwise F-tests between histotype IC50 for Bleomycin (**a**) and Vorinostat (**b**). **c**-**d** Measured vs Predicted IC50 using DEGs for Bleomycin (**c**) and Vorinostat (**d**). **e**-**f** Measured vs. Predicted IC50 for Hist models in Bleomycin (**e**) and Vorinostat (**f**). Each symbol color combination indicates a different histotype

Several of the drugs cell line predictions of the same histotype tended to cluster together as illustrated with vorinostat (Fig. 5d.) suggesting that histotype might be predictive of drug response. In order to ascertain if there was an actual differential drug response between histotypes, we performed pairwise F-tests between drug responses categorized by histotype. The number of significant pairwise comparisons ranged from a low of 5.1% for bleomycin (Fig. 5a.) to 52.6% for vorinostat (Fig. 5b.) with an average of 24.1%. Furthermore, the Spearman correlation between the percentage of significant F-tests and the average Spearman correlation for the two control models was 0.85 and 0.88 for NLSVR and 0.84 and 0.86 for PCR on CTR1 and CTR2 datasets respectively.

To establish the influence of histotype on model performance it had to be shown that histotype could predict drug response, and that any feature selection methods yielded features with the equal ability to distinguish one histotype from another. To accomplish this a 55 dimensional feature matrix was developed to encode cell line identity to one of the 55 possible histotypes represented in the data using one hot encoding. Then using this feature matrix NLSVR and LSQR were applied to predict drug response. In both NLSVR and LSQR there was a significant drop in the average Spearman correlation, 0.2193 for NLSVR and 0.2218 for LSQR. However, 61–62% of the models gave significant correlations in both NLSVR and LSQR (Fig. 4c and d). For several drugs, such as bortezomib, cisplatin, docetaxel, gemcitabine, methotrexate, paclitaxel, and vorinostat gave comparable results. Whereas for others, such as bleomycin, doxorubicin, 5-fluorouracil, and SN-38 substantially lower correlations were obtained. The ability of histotype to predict response is best illustrated between bleomycin (Fig. 5a, c, and e.) and vorinostat (Fig. 5b, d, and f.). Bleomycin has minimal differential drug response between histotypes (Fig. 5a.) as a result, when given nothing but histotype as input the model will have a tendency to predict the average IC50 values of a given histotype. In the case of bleomycin the average IC50 values of different histotypes do not exhibit a great amount of variability and thus the predictions collapse to the overall average of the data (Fig. 5e.). Alternatively, for drugs such as in vorinostat, the histotype average IC50 values exhibit a greater amount of variability (Fig. 5a.) and as a result this variability is reflected in the predictions (Fig. 5f.). Furthermore, the variability of average histotype responses is roughly captured when the features are reduced to only indicators of histotype in drugs such as vorinostat (Fig. 5d.) where this is absent in bleomycin (Fig. 5c.)

To explore the ability of a given set of features to identify histotype we used k-means clustering to cluster the cells into one of 55 groups and then used cluster entropy, S_c,_ to quantify the consistency of which cells of the same histotype were placed in the same cluster. A pairwise non-parametric Wilcoxen paired T-test showed that there was no significant difference between DEG, CTR1, and CTR2 genes, and while S_c_ for NO FS was statistically significant it is not apparent if there is a meaningful difference as the average absolute difference was only 8.5% (Fig. 4e.). Additionally, while BS, BC, and MRMR had higher average S_c,_ 100% of BS models, 98.8% of MRMR models, and 86.7% of BC models clustered by histotype better than a randomized model. Therefore, the data suggests that the predictive ability of the model is partially dictated by the ability of a set of features to recognize similar histotypes as well as the variability between drug responses between histotypes.

### Model performance, number of features, histotype recognition

To determine how the number of genes affected the performance, if genes statistically linked to drug response became a bigger factor as the number of features decreased, and how both of these affected the ability to cluster cells based on histotype, we constructed models for both NLSVR and PCR using 10, 55, 250, 500,1000 randomly selected features from DEGs, CTR1, or CTR2 as well as performing k-means clustering.

**Fig. 6:**
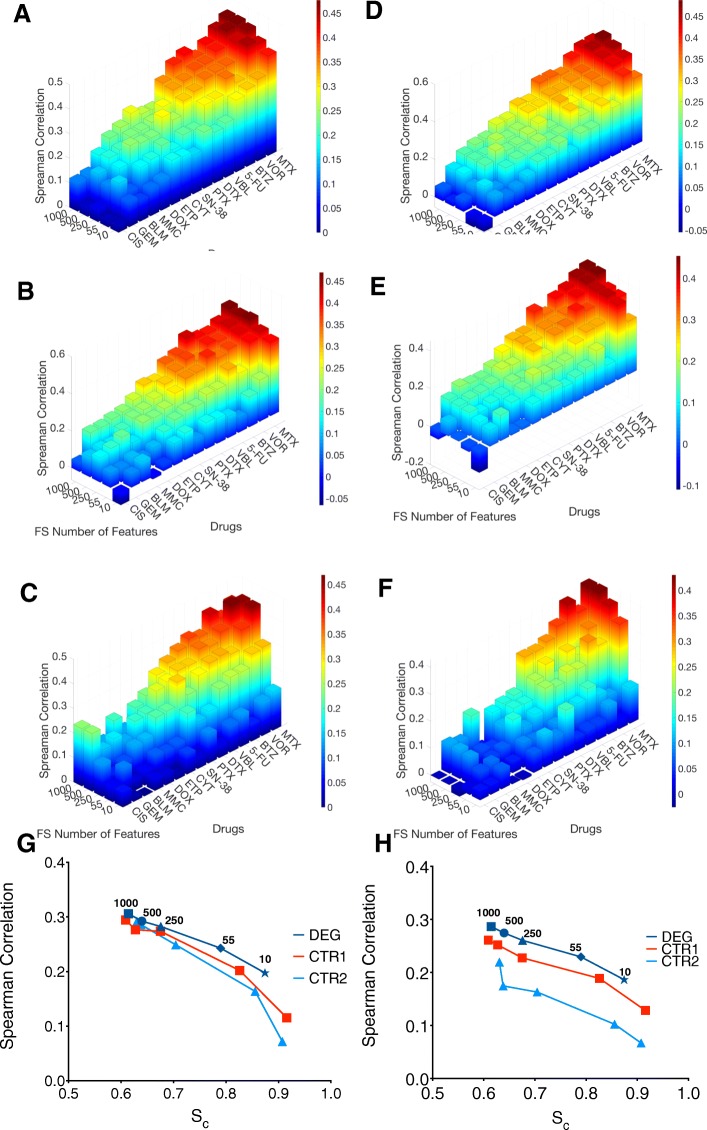
Model performance and number of features. Average Spearman correlations for all 15 drugs as a function of features used for NLSVR DEG (**a**), NLSVR CTR1 (**b**), NLSVR CTR2 (**c**), PCR DEG (**d**), PCR CTR1 (**e**), PCR CTR2 (**f**). average S_c_ vs average Spearman correlation with each symbol representing the number of features used for NLSVR (**g**) and PCR (**h**)

As expected, a decrease in overall performance was observed as the number of features decreased. However, the magnitude of performance drop was considerably different depending on the feature selection method. In NLSVR the performance of DEG models dropped by 36%, CTR1 models dropped by 61%, and CTR2 models dropped by 71% (Fig. 6a, b, c, and g.). Likewise, PCR models decreased by 35.2, 51 and 70% in DEG, CTR1and CTR2 models respectively (Fig. 6d, e, f, and h). Furthermore, DEG feature selection in both PCR and NLSVR models are reasonably robust down to 250 features, with NLSVR exhibiting only a 7.5% difference and PCR only 9.1% at 250 features compared to 1000 features (Fig. 6a, d, g, h). Likewise, in CTR1 models, only a 7.1% decrease in NLSVR and 11.9% decrease in PCR (Fig. 6b, e, g, and h). CTR2 models exhibited a decrease approximately twice as great (14.4% NLSVR and 26% PCR) as that seen with DEGs or CTR1 features (Fig. 6e, f, g, and h).

For NLSVR models the difference in S_c_ is minimal down to 500 features for all three feature selection methods and down to 250 genes for DEG and CTR1 features. As a result, there is not a substantial difference in performance down to 500 or less features (Fig. 6g). Additionally, for DEG and CTR1 genes the difference in S_c_ is only about 7% going from 1000 to 250 features (Fig. 6g). The drop in S_c_ reflects a loss in a given set of features to identify histotype and is coupled directly to a loss in performance. Furthermore, substantial performance differences between DEG and CTR1 features began to occur at 55 genes which is also marked by a substantial increase in S_c_ between DEG and CTR1 genes, and begins happening at 250 CTR2 features (Fig. 6g). PCR models exhibit a clear discrepancy between all three feature selection methods; however, as the difference in S_c_ increases between methods the difference in performance grows as well consistent with the idea that S_c_, and therefore, histotype, has a substantial influence on model performance (Fig. 6h). Lastly, as the number of features is dropped considerably, DEG features maintain more histotype specificity which suggest that features which are highly correlated to drug response are also highly correlated with histotype.

### Comparison with DREAM

The DREAM-NCI project assessed the performance of each model using a modified concordance index which they called the weighted-probability concordance index (wpc-index). Given the vast diversity of models evaluated we wanted to determine if the models we used were comparable using the wpc-index. The average wpc-index was 0.576 with a range from 0.552 to 0.582 for NLSVR and 0.569 for PCR ranging from 0.552 and 0.58 (Table 5). For Both NLSVR and PCR methods the models with the highest wpc-index were DEG models while the lowest performing model were the histotype-only models. The Spearman correlation of the wpc-index with average Spearman correlation was 0.9833 (*p* = 0) for NLSVR models and 0.95 (p = 0) for PCR models. The top models in the DREAM-NCI paper have wpc-index scores of 0.583, 0.577, and 0.57, and the minimum score was calculated to be 0.485. While, based on wpc-index, we had no models out-perform the top performing model, four NLSVR models had wpc-index scores that would place them in second place, and all, with the exception of the HIST model, scored within the top three. Likewise, PCR, had two models that scored above the second place model and six that placed above the third place model Table 5.

**Table 5:** WPC Index

FS Method	NLSVR	PCR	NLSVR NSCLC-AD	p NSCLC-AD
NO FS	0.581	0.578	0.528	0.009
DEG	0.582	0.579	0.535	0.001
CTR1	0.581	0.576	0.519	0.056
CTR2	0.576	0.559	0.508	0.248
MRMR	0.579	0.575	NA	NA
BS	0.58	0.576	NA	NA
BC	0.575	0.571	NA	NA
Hist	0.552	0.552	NA	NA
Random Control	NA	0.498	NA	NA

WPC Index for NLSVR and PCR models as well as values for non small cell lung cancer adenocarcinoma (NSCLC-AD) in select drugs. The-value for NSCLC-AD was calculated by 3000 random permutations of the test data to construct a null distribution. For example, there is a .01% chance of obtaining a higher WPC score randomly for NLSVR DEG models on NSCLC-AD. Note that a wpc score cannot be calculated for the random control for NLSVR due to a variance of 0 which results in division by 0 incalculated wpc scores

The DREAM-NCI project consisted only of a single cancer histotype (breast) and thus, the histoytpe phenomena driving the performance of our models is not a contributing factor in their models. The testing set for the DREAM-NCI project consisted of 18 cells lines, the number of cells of a single histotype did not exceed 20 and was often below 10 for any test split. However, non-small cell lung carcinoma adenocarcinoma (NSCLC-adenocarcinoma) was represented with 10 or greater cells in 10 of the 15 drugs and 43% of the total testing data sets. Thus, in order to gauge if the models were picking up some cell specific drug response within a histotype we used the WPC index to score DEG, CTR1, CTR2, and No FS models. Compared to wpc scores for our pan-cancer models and several models in the DREAM project the WPC index was smaller ranging from 0.5346 for DEG Models to 0.5084 for CTR2 models (Table 3). Considering the variable number of cell lines for each dataset, we assessed the significance by creating a null distribution of 3000 randomly constructed permutations of the modeled data. DEG and No-FS had wpc scores that significantly differed (*p* < 0.05) from what would be expected by random permutation with a wpc value of 0.5. This suggests models which include genes relevant to drug response have some ability to pick up variability in individual histotypes, while genes with no apparent significantly statistical relationship with drug response fail to pick up that variability indicated by having a wpc score consistent with a random permutation of the data.

## Modeling the NCI60

**Fig. 7:**
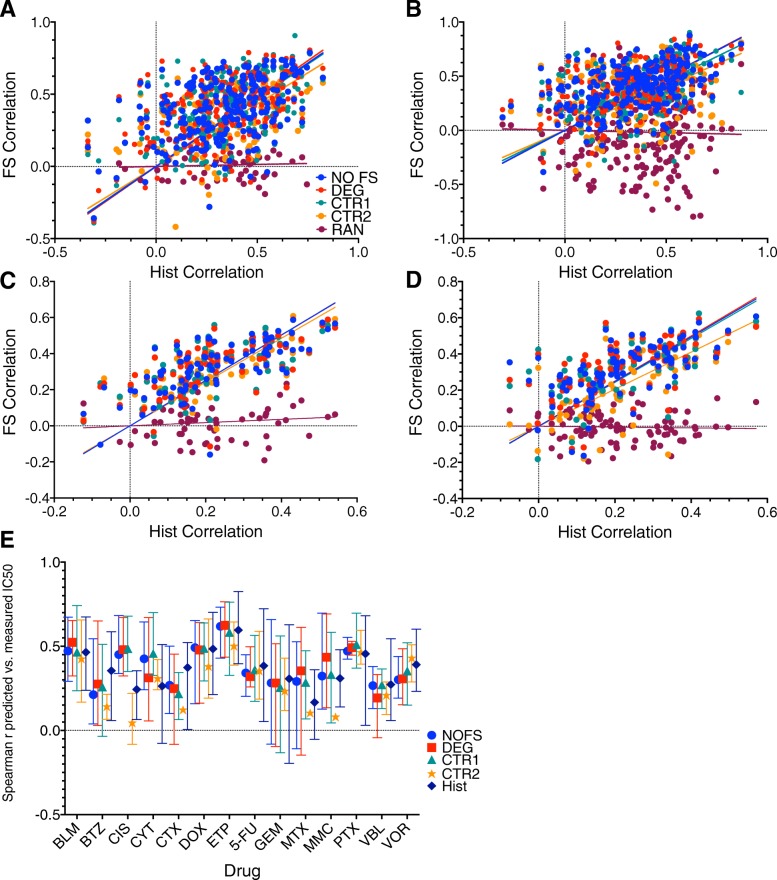
NCI60 models vs the GDSC models: **a**-**d** Spearman correlations for a given feature selection method (FS correlation) verses Spearman correlations for one hot encoded histotype models for NCI60 NLSVR (**a**), NCI60 PCR (**b**), GDSC NLSVR (**c**), GDSC PCR (**d**). **e**. NCI60 models for the 14 drugs with data both in the GDSC and NCI60

The NCI60 results were highly variable due to the low sample size, but many of the trends that emerged in the GDSC were also evident in the NCI60, mainly, there was not a significant difference in performance between NLSVR and PCR, and minimal difference between selected features (NOFS: 0.4, DEG: 0.403, CTR1: 0.399, CTR2: 0.351) for SVR and (NOFS: 0.412, DEG: 0.406, CTR1: 0.382, CTR2: 0.35) for PCR. One of the more interesting points is that models performance still has a significant relationship with histotype as evidenced by significant correlation histotype models and models constructed with genomic feature with correlation ranging 0.4114 to 0.4576 for NLSVR and 0.2988–0.4547 for PCR (Fig. 7 A. and B.) This is relationship is even stronger in the GDSC (NLSVR:0.6878–0.7341, PCR: 0.663–0.733) due to the increased number of cell lines and histotypes (Fig. 7 C. and D.). It is also important to note that the GDSC and NCI60 share 38 cell lines, however, the range is smaller in the drugs we modeled [7–30]. For the 14 common drugs with data in the NCI60 and GDSC only 7 had significant correlations in drug response for identical cell lines.

## Additional Models

In addition to the models discussed we implemented an Elastic Net model in the GDSC, which did not show any significant difference in results. The results of this model can be found in the Additional file 2 Furthermore, we further explored how sampling techniques, and how the non-uniform representation of histotypes possibly could impact results in a select subset of models (Additional file 2).

## Discussion

The development of molecular tools allows for a unique look into the molecular nature of cancer inspiring a community effort to collect data with the potential for significant clinical impact. Given the complexity and amount of data that is available and continues to be generated, computational approaches are necessary to fully utilize the available information present in the data. As these computational techniques become more advanced, a basic understanding of the factors that influence model performance are essential. We have taken a systematic approach to characterize the influence of basic model complexity in terms of the linearity of the the model and basic CBF feature selection methods to predict in vitro drug response across a number of cancer cell lines in the GDSC and NCI60. Our results suggest that the complexity of the model and the method of feature selection have marginal effects on the performance of the model with performance largely dictated by the relationship between histotype and drug response [See Additional file 2: Figure S5].

With the exception of ANN, it is not straightforward to establish which model is superior as it is certainly reasonable that more thorough parameter optimization and different data splits might lead to fractional improvements for one method over others changing the relative rank of each model while having insignificant meaningful quantitative gain. Likewise, there is no substantial gain by eliminating features that do not significantly correlate with drug response. The control experiments (CTR1 and CTR2) as well as the histotype models suggest that histotype has a major influence on predictive capability of model. More rigorous criteria as imposed by Bonferroni correction and bootstrap methods tend to decrease model performance slightly and this is accompanied by a similar decline in selected features ability to cluster cell lines by histotype as evidenced by a larger S_c._ Attempts to remove redundancy in features using MRMR results in higher S_c_ values suggesting that the histoytpe signal can be somewhat mitigated by removing redundant features but is also accompanied by a decrease in overall performance. Furthermore, even at 500 random features a diffuse histotype signature is maintained which maintains the majority of drug-response information sufficient for producing predictive models.

Our best performing model consisted of Support Vector Regression using a radial basis function. However, the improvement over the best performing linear model, PCR, was only a 3.8% increase in average Spearman correlation. Likewise, the DREAM competition concluded that non-linear models performed slightly better than linear models; however, the performance increase between their top non-linear model and top linear model was only 1.5% with several linear models performing better than many other non-linear models developed [21]. Artificial neural networks performed consistently the worst, for comparison, Menden et al. used ANN to build predictive drug response models for 608 cell lines and 111 drugs in the GDSC using genomic and chemical properties of drugs reporting an overall Pearson correlation of 0.85 across all drugs. However, the individual drug correlations ranged roughly from − 0.15 to 0.5 similar to the results we achieve [49]. The discrepancy in Menden’s work between the overall correlation and the individual drug correlations is most likely due to the spread of IC50’s across drugs as different drugs have distinct ranges of IC50 values, this can clearly be seen in (Fig. 3 B-E) demonstrating how inherent data structure, i.e. different ranges of drug response for different drugs, can introduce artifacts that can potentially affect both the construction and analysis of models. Other modeling strategies in the GDSC that have focused on targeted agents have produced average spearman correlations slightly higher, (approximately 10%) [50] while models incorporating Bayesian components have led to significantly lower average Spearman correlations (around 50%) [51].

Our results show that neither the linearity of the regression method nor features used have a strong influence on performance with the single most influencing factor being the identity of the drug. Furthermore, this is consistent across multiple data-bases and over many cytotoxic agents despite inconsistencies seen among cell lines shared by the GDSC and NCI60 that could prove a barrier to using the GDSC to train and validate a model and test on the NCI60 or likewise the NCI60 to train and validate testing on the GDSC. This phenomenon results from the tendency that cancers from the same histological background respond similarly to certain drugs. This is reflected in our results; predictive outcomes can be achieved in most drugs simply by identifying the histotype. Consequently, any gene set that has the ability to differentiate histotype also can generate predictive models as demonstrated with our control models. Often the identification of histotype is an essential step into determining specific approaches to successful treatment, clearly not all cancers of the same histotype respond precisely the same to a given drug. The range of responses might have important consequences when it comes to determining effective PKPD parameters for clinical applications. Furthermore, with respect to modeling, this “histotype” effect potentially shields features that have significant predictive capability across all histotypes where the signal to noise ratio is significantly less compared to features that have strong associations with histotype.

Several successful models have been built to classify tumors histologically using genomic profiling [3, 5, 52, 53] demonstrating the ability of statistical learning techniques to learn tissue specific features. Thus, given the differential drug response of cancers with similar histological background the prediction of response loosely defaults to a classification exercise. This simultaneously presents opportunity and challenge. Knowing the histotype, therefore, gives a significant amount of information about the drug response. However, histotype accounts for a large amount of genomic variation as well as variability in drug response. Therefore, feature selection results in the convolution of three possible categories: features that account for variability in histotype having no influence on actual drug response, features that account for variability in histotype and drug response, and lastly features in which variability is exclusively a result of drug response. This is a challenging task, filter methods, such as CBF and mutual information, tend to pick more robust signals associated with drug-histotype interactions. The ability to extract drug response within a histotype and then leverage that information across histotypes, such as ensemble methods, might be a reasonable approach. For example, a filter based feature selection method could be applied on each histotype independently then those features present in all histotypes could be pooled. Additionally, a multiple kernel learning (MKL) method where each individual kernel is applied to a distinct histotype might be an effective way to pool multiple histotype based models into a more generalized pan cancer model. However, number of samples of each histotype in most databases, such as the GDSC, could be a limiting factor for producing robust features in a filter based method or parameter optimization in a MKL setting. Databases such as the NCI60, GDSC, Cancer Therapeutics Response Portal (CTRP), and Cancer Cell Line Encyclopedia (CCLE) would provide for a broader diversity of data. However, several studies have shown inconsistency in drug response among cell lines derived from the same source [54–56].

## Conclusion

The ultimate goal for these types of predictive models is to become a clinical tool that practitioners can utilize to improve the treatment of cancer patients or to inform clinical trials. While the jump from an in vitro cancer cell line to a tumor and then eventually a patient is a considerable progression these in vitro based experiments certainly add insight to the problem. Previous studies that have leveraged in vitro data to inform tumor based predictions have approached drug response as a binary variable, sensitive or resistant [15, 16]. However, in such an approach valuable quantitative insight might be lost that could be critical to successful clinical applications. For example, an in vitro cell line might exhibit an IC50 that is much lower in comparison to other cell lines, implying sensitivity, but the concentration of drug needed to achieve a comparable exposure in a patient might not be reasonable due to pharmacokinetic or toxicity constraints. Thus, to more effectively use cell line drug exposure the ability to first accurately capture in vitro drug response is critical. What our models suggest is that similar pan-cancer cell based models might over emphasize a relationship between histotype and drug response thus could be misleading when applying such techniques to tumor data by effectively only capturing a broad histotype response failing to be applicable to more inter-tumor variability. Therefore, it is paramount that drug-histotype response is considered to improve model performance and utility.

Biological systems are inherently complex, noisy, and high dimensional which makes modeling their behavior a difficult task. Statistical learning allows for the extraction of valuable insights from large sets of data without direct knowledge of the intrinsic mechanisms that are influencing the properties of the system. For this reason, statistical learning provides several tools that are directly applicable to cancer diagnosis and treatment and it has been an active area of cutting edge research in cancer biology, mathematics, statistics, and computer science. Therefore, as the field moves forward it is absolutely imperative to understand how fundamental modeling considerations influence model performance on large complex biological datasets. Systematic approaches with well thought out control experiments are paramount to fully understand the complexities that arise when considering different modeling strategies.

## Additional files

Additional file 1: Spearman correlations and MAD values of the 39 Drugs modeled in the NCI60. (XLSX 248 kb)

Additional file 2:  This file incudes data for additional feature selection methods and elastic net models as well as model results reported as mean absolute difference. Additionally this file includes discussions about cross validation and histotype distribution. (DOCX 4430 kb)

Additional file 3: *P* values for information entropy reported in Figure 4e. (XLSX 28 kb)

Additional file 4: Spearman correlations for ANN models reported in Results section A. and B and illustrated in Fig. 3. (XLSX 26 kb)

Additional file 5: MAD for ANN models reported in Results section A. and B. (XLSX 18 kb)

Additional file 6: Spearman correlations for SVRLN models reported in Results section A. and B and illustrated in Fig. 3. (XLSX 24 kb)

Additional file 7: MAD for SVRLN models reported in Results section A. and B. (XLSX 18 kb)

Additional file 8: The file includes the values of the Spearman correlation for PCR models corresponding to Figs. 3, 4, and 5. (XLSX 86 kb)

Additional file 9: The file includes the MAD values for PCR models reported in results section A, B, C, and D. (XLSX 44 kb)

Additional file 10: Spearman Correlations for NLSVR DEG models from 10 to 1000 genes presented in figure for section E and Fig. 6. (XLSX 38 kb)

Additional file 11: MAD values for DEG NLSVR models discussed in section E. (XLSX 26 kb)

Additional file 12: Spearman Correlations for PCR DEG models from 10 to 1000 genes presented in figure for section E and Fig. 6. (XLSX 39 kb)

Additional file 13: MAD values for PCR DEG models discussed in section E. (XLSX 19 kb)

Additional file 14: Spearman Correlations for NLSVR CTR1 models from 10 to 1000 genes presented in figure for section E and Fig. 6. (XLSX 38 kb)

Additional file 15: MAD values for NLSVR CTR1 models discussed in section E. (XLSX 26 kb)

Additional file 16: Spearman Correlations for PCR CTR1 models from 10 to 1000 genes presented in figure for section E and Fig. 6. (XLSX 39 kb)

Additional file 17: MAD values for PCR CTR1 models discussed in section E. (XLSX 25 kb)

Additional file 18: Spearman Correlations for NLSVR CTR2 models from 10 to 1000 genes presented in figure for section E and Fig. 6. (XLSX 36 kb)

Additional file 19: MAD values for NLSVR CTR2 models discussed in section E. (XLSX 26 kb)

Additional file 20: Spearman Correlations for PCR CTR2 models from 10 to 1000 genes presented in figure for section E and Fig. 6. (XLSX 38 kb)

Additional file 21: MAD values for PCR CTR2 models discussed in section E. (XLSX 25 kb)

Additional file 22: This file includes the values of the Spearman correlation for NLSVR models corresponding to Figs. 3, 4, and 5. (XLSX 67 kb)

Additional file 23: This file includes MAD values corresponding to Figs. 3, 4, and 5. (XLSX 44 kb)

Additional file 24: The number of cell lines from each histotype for each drug each drug. (CSV 3 kb)

Additional file 25: Spearman correlations and MAD values for 5 fold nested cross validation for NLSVR models discussed in the supplementary materials. (XLSX 78 kb)

Additional file 26: Spearman correlations and MAD scores using comparing tissue stratified and non-stratified models for a subset of the data as discussed in supplementary materials. (XLSX 68 kb)

## Abbreviations

ANN: Artificial Neural Networks
BS: Boot-Strapped
DEG: Differentially expressed genes (probes)
GDSC: Genomics for Drug Sensitivity in Cancer
Hist: Histotype
IC50: The half maximal inhibitory concentration
NLSVR: Non-linear Support Vector Regression
PCR: Principle Components Regression
S_n_: Cluster Entropy
SVRLN: Linear Support Vector Regression
WPC Index: Weighted-probability concordance index
