# Supplementary material for: A systematic analysis of genomics-based modeling approaches for prediction of drug response to cytotoxic chemotherapies
Source: BMC Med Genomics. 2019 Jun 17;12:87. doi: 10.1186/s12920-019-0519-2 (PMC6580596; doi:10.1186/s12920-019-0519-2)
Supplement: Supplementary file 2 — This file incudes data for additional feature selection methods and elastic net models as well as model results reported as mean absolute difference. Additionally this file includes discussions about cross validation and histotype distribution. (DOCX 4430 kb) [file 12920_2019_519_MOESM2_ESM.docx]

**Supplementary Materials.**

**Contents:**

1. **Mean Absolute Difference compared to Spearman Correlation.**
2. **Additional feature selection methods**
3. **Linear Analysis of Histotype**
4. **Elastic Net Models**
5. **Cross Validation Strategies**
6. **Differences within Histotype Distributions**
7. **Additional figures**

**Mean Absolute Difference Results:**

The Spearman correlation is a relative measure that is particularly useful in describing general trends in the data, however, other measures such as root mean squared error (RMSE) and mean absolute difference (MAD) are better at quantifying the overall accuracy of the model. Here we show that the same quantitative patterns are captured by Spearman correlations are also present in MAD. Average MAD values accompanied with the corresponding average spearman correlations, and the Spearman correlation between MAD values and Spearman correlations for each model are reported in supplementary table 1. Average MAD values ranged from a minimum of 0.881 to 1.391 with a mean value of 0.931 and standard of deviation 0.067. Comparatively, the average Spearman correlations ranged from -0.011 to a maximum value of 0.334 with a mean of 0.253 and a standard of deviation of 0.083. As with with Spearman correlation average MAD values gave relative performance as NLSVR>PCR>SVRLN>ANN. The MAD value of a model was strongly negatively correlated to a model Spearman correlation (Sup. Figure 1 C). The correlation between model average MAD scores and average model Spearman correlations also had a strong negative correlation -0.8418 (Sup Figure 1 B.). MAD scores generally reflect the behavior that drugs with higher correlations generally have lower MAD scores (Sup figure 1 B.). However, within each drug there is not a strong correlation between a given datasets correlations and their MAD scores (Sup Figure 1 B.). This can be explained by the influence of histotype in predictions. For example, for drugs such as vorinostat, bortezomib, and methotrexate the high correlations are attributed to correctly identifying the association between histotype and drug response, however, for these drugs, within a given histotype the predicted values do not necessarily have a strong correlation with the experimental values. Therefore, within a histotype the MAD score is more a reflection of the variability within each histotype that results as noise around an average IC50 for that histotype. Interestingly, for drugs which show smaller response to histotype, bleomycin, doxorubicin, cytarabine, and mitomycin slight negative correlation can be seen between MAD and Spearman Correlation (Sup. figure 1 B). Additionally, the range of experimental IC50’s can have an effect. As an extreme example Cisplatin, which with respect to Spearman correlation, performs the worst has the lowest MAD values. This results from the fact that the range of IC50’s for Cisplatin is much smaller than the other drugs, which might also explain why it is particularly hard to yield good predictions. Therefore, variability of the given dataset plays a role in MAD scores which is minimal with respect to Spearman correlation.

|  | AVG r | AVG MAD | r vs MAD | p |
| --- | --- | --- | --- | --- |
| LSQR_HIST_ONLY | 0.206600941 | 0.99311685 | -0.789215686 | 0.000165507 |
| NNet1_DEG | 0.268050903 | 1.001504775 | -0.825635314 | 4.39E-22 |
| NNet1_Limma | 0.265734239 | 1.031461462 | -0.684458844 | 7.06E-13 |
| NNet1_None | 0.170836501 | 1.39071894 | -0.519894705 | 4.02E-07 |
| PCR_BC_DEG | 0.299126885 | 0.892740612 | -0.742411924 | 2.16E-15 |
| PCR_BS_DEG | 0.328409628 | 0.89976564 | -0.74097398 | 7.75E-16 |
| PCR_BS_hist | 0.231715541 | 0.927316692 | -0.778454996 | 2.92E-18 |
| PCR_CTR1 | 0.327679648 | 0.89026602 | -0.79437076 | 1.94E-19 |
| PCR_CTR1_10 | 0.145384873 | 0.94669059 | -0.503735952 | 1.04E-06 |
| PCR_CTR1_1000 | 0.282793893 | 0.907468456 | -0.77946745 | 2.47E-18 |
| PCR_CTR1_250 | 0.242837144 | 0.923793142 | -0.741905437 | 6.83E-16 |
| PCR_CTR1_500 | 0.264982445 | 0.917180188 | -0.738787081 | 1.04E-15 |
| PCR_CTR1_55 | 0.204242863 | 0.935326004 | -0.731861901 | 2.61E-15 |
| PCR_CTR2 | 0.259641397 | 0.913025063 | -0.777908272 | 3.19E-18 |
| PCR_CTR2_10 | 0.069563485 | 0.958145959 | -0.11588539 | 0.293840292 |
| PCR_CTR2_1000 | 0.235427127 | 0.924755282 | -0.707765516 | 5.17E-14 |
| PCR_CTR2_250 | 0.176621455 | 0.939921685 | -0.608909588 | 7.98E-10 |
| PCR_CTR2_500 | 0.187357689 | 0.93702075 | -0.67718943 | 1.52E-12 |
| PCR_CTR2_55 | 0.107959474 | 0.950592666 | -0.267712868 | 0.013817181 |
| PCR_DEG | 0.338371044 | 0.89577949 | -0.807168168 | 1.83E-20 |
| PCR_DEG_10 | 0.203332115 | 0.939005307 | -0.677533664 | 1.47E-12 |
| PCR_DEG_1000 | 0.302904109 | 0.91008021 | -0.804576288 | 3.00E-20 |
| PCR_DEG_250 | 0.274990668 | 0.922032562 | -0.732226385 | 2.49E-15 |
| PCR_DEG_500 | 0.292483261 | 0.909676385 | -0.761587527 | 4.08E-17 |
| PCR_DEG_55 | 0.249617233 | 0.928768805 | -0.734717019 | 1.79E-15 |
| PCR_DEG_HIST | 0.321946531 | 0.912223626 | -0.778758732 | 2.78E-18 |
| PCR_Limma | 0.297227647 | 0.902728419 | -0.722689076 | 8.44E-15 |
| PCR_MRMR | 0.315486982 | 0.92277003 | -0.747777665 | 3.03E-16 |
| PCR_None | 0.336920624 | 0.886850982 | -0.784732206 | 1.03E-18 |
| PCR_RAN_CTR | -0.011832006 | 0.967311497 | 0.054247241 | 0.624066277 |
| SVRLN_DEG | 0.2840744 | 0.967909486 | -0.801761669 | 5.07E-20 |
| SVRLN_Limma | 0.279914537 | 0.937635103 | -0.685896527 | 6.05E-13 |
| SVRLN_None | 0.314662929 | 0.922962809 | -0.79763086 | 1.08E-19 |
| SVR_BC_DEG | 0.311197619 | 0.890135989 | -0.788775971 | 2.26E-18 |
| SVR_BS_DEG | 0.319205813 | 0.855917246 | -0.756 | <0.0001 |
| SVR_BS_hist | 0.323632754 | 0.893051569 | -0.784266478 | 1.11E-18 |
| SVR_CTR1 | 0.347084298 | 0.881359019 | -0.812250683 | 6.84E-21 |
| SVR_CTR1_10 | 0.128314394 | 0.947990492 | -0.428895414 | 4.69E-05 |
| SVR_CTR1_1000 | 0.31239602 | 0.896025151 | -0.813769363 | 5.06E-21 |
| SVR_CTR1_250 | 0.283867927 | 0.906474413 | -0.725442948 | 5.96E-15 |
| SVR_CTR1_500 | 0.288746646 | 0.907812295 | -0.759542371 | 5.54E-17 |
| SVR_CTR1_55 | 0.210776307 | 0.927963843 | -0.722527083 | 8.61E-15 |
| SVR_CTR2 | 0.328040711 | 0.904836275 | -0.749559583 | 2.35E-16 |
| SVR_CTR2_10 | 0.072550415 | 0.95671404 | -0.401781918 | 0.000151772 |
| SVR_CTR2_1000 | 0.294807627 | 0.898299147 | -0.687366115 | 2.63E-12 |
| SVR_CTR2_250 | 0.25805736 | 0.901661149 | -0.760345667 | 4.38E-16 |
| SVR_CTR2_500 | 0.28915211 | 0.924286006 | -0.722324592 | 8.83E-15 |
| SVR_CTR2_55 | 0.17142137 | 0.941638918 | -0.647949782 | 2.70E-11 |
| SVR_DEG | 0.348704324 | 0.885688225 | -0.79698289 | 1.21E-19 |
| SVR_DEG_10 | 0.209667666 | 0.936247036 | -0.66854308 | 3.68E-12 |
| SVR_DEG_1000 | 0.319370711 | 0.896286971 | -0.766690291 | 1.88E-17 |
| SVR_DEG_250 | 0.299125501 | 0.904835421 | -0.786736863 | 7.32E-19 |
| SVR_DEG_500 | 0.310820283 | 0.896687404 | -0.804961021 | 2.79E-20 |
| SVR_DEG_55 | 0.258337555 | 0.919247829 | -0.696770274 | 1.83E-13 |
| SVR_DEG_HIST | 0.333098198 | 0.899440672 | -0.809415815 | 1.19E-20 |
| SVR_HIST_ONLY | 0.229659101 | 0.930236405 | -0.784894199 | 1.00E-18 |
| SVR_Limma | 0.316195531 | 0.895748638 | -0.754621849 | 1.14E-16 |
| SVR_MRMR | 0.326862919 | 0.905420517 | -0.76820897 | 1.49E-17 |
| SVR_None | 0.345708834 | 0.881855077 | -0.780074922 | 2.24E-18 |
| SVR_RAN_CTR | 0.008792232 | 0.967722401 | -0.123855092 | 0.363112309 |

Supplementary Table 1: Each models average Spearman Correlation, Average MAD score, and the correlation between the average Spearman correlation and MAD along with the associated P value

**Additional Feature Selection Methods:**

The relationship between histotype and drug response dictated both model performance and feature selection. Selected features tended to reflect this relationship, presumably masking features that could account for subtler differences in drug response. Therefore, an approach that could balance variability due to histotype with variability more specific to drug response might allow for better model performance. This suggested that the problem could be addressed either by muting features selected purely based on histotype or adding additional histotype specific covariates to account for histotype variability while allowing genomic covariates to account for drug variability independent of histotype.

In general, histotypes were not equally represented in each dataset. This becomes problematic when two or more histotypes that are disproportionally represented in the training data have dramatic differences in drug response which results in the selection of genetic features which might show differential expression as a result of histotype rather than drug response. Presumably this effect could be mitigated by using a more uniform representation of histotypes during feature selection. However, by specifically curating datasets such that each histotype had equal representation introduced the possibility that selected features would be biased towards the makeup of the individual choice of dataset. We addressed these issues by constructing 50 subsets of the data containing one sample from a histotype and then taking genes that were significantly correlated (P<0.05) in at least half the subsets (Boot Strapped by histotype: “BS Hist”). As expected features were dramatically reduced ranging from 99.99 to 88.65 percent with an average decrease of 98.6 percent. This method resulted in the lowest average Spearman correlation of all feature selection methods, 0.304 for NLSVR (Sup. Figure 2 A) and 0.216 for PCR (Sup. Figure 2 B). S_c_ also was significantly higher than DEG and no feature selection, consistent with the observation that for a given set of features a decreased ability to discriminate samples by histotype results in a decrease in performance. Analysis of the selected genes showed that genes which met the criteria for selection still showed significant variability according to histotype, thus again these models defaulted to fitting an average histotype IC50.

Alternatively, by adding an additional histotype specific variable we attempted to break the regression into two different groups of terms:

$$\boldsymbol{Y}(h,\boldsymbol{G})=f\left( h \right)+f(\boldsymbol{G})$$

where $\boldsymbol{Y}$ is the drug response, $h$ is the histotype, and $\boldsymbol{G}$ is the gene expression matrix. This was accomplished by combining the feature matrix for the histotype models and the DEG expression values scaled from zero to one to avoid scaling issues. The motivation behind this was to place the variability strictly due to histotype on the histotype term allowing non-redundant variability independent of histotype to play a more active role in predicting drug response. However, the addition of histotype specific variables resulted in an overall decrease in average performance, 0.319 for NLSVR and 0.306 for PCR (Sup figure 2 A and B). The failure of this method further suggests that sources of genomic variability that are small compared to the variability resulting from histotype are effectively treated as noise which both NLSVR and PCR aim to minimize by regularization in NLSVR or the elimination of components in PCR.

**Linear Analysis for histotype:**

We have demonstrated that the relationship between histotype and IC50 is a major driver of model performance. Given this relationship, it is reasonable to ask how comparable are our models to a linear model where the predicted IC50 is simply a function of the average IC50 value for a given histotype with the addition of random noise:

$$Y=\beta_{1}*Z+N(0,\sigma_{1})$$

where $Y$ is the predicted IC50, $Z$ is a random variable given by the average IC50 of a random sample of cells of a single histotype, $\beta_{1}$ is a constant, and $N(0,\sigma_{1})$ is a normal distribution with mean 0 and variance $\sigma_{1}$. Additionally, the experimental IC50 values, $X$, can also be measured similarly:

$$X=\beta_{2}*Z+N(0,\sigma_{2})$$

it can be shown that $\rho_{XY}=\rho_{XZ}\rho_{YZ}$ where $\rho_{NM}$ is the Pearson correlation of $N$ and $M$as follows:

by the definition of covariance

$$cov\left[ X,Y \right]=E\left[ \left( X-E\left[ X \right] \right)*\left( Y-E\left[ Y \right] \right) \right]$$

$$cov\left[ X,Y \right]=E[XY-YE\left[ X \right]-XE\left[ Y \right]+E\left[ X \right]E\left[ Y \right]]$$

$cov\left[ X,Y \right]=E\left[ XY \right]-E\left[ YE\left[ X \right] \right]-E\left[ XE\left[ Y \right] \right]+E[E\left[ X \right]*E\left[ Y \right]]$ [1]

the expectation value of $X$ and $Y$ is given by

$E\left[ X \right]=E\left[ \beta_{1}*Z+N\left( 0,\sigma_{1} \right) \right]=\beta_{1}E[Z]$ [2]

likewise

$E\left[ Y \right]=\beta_{2}E[Z]$ [3]

similarly

$E\left[ YE\left[ X \right] \right]=E\left[ XE\left[ Y \right] \right]=E\left[ X \right]E\left[ Y \right]=\beta_{1}\beta_{2}{E[Z]}^{2}$ [4]

$$E\left[ XY \right]=E[\left( \beta_{1}*Z+N\left( 0,\sigma_{1} \right) \right)*\left( \beta_{2}*Z+N\left( 0,\sigma_{2} \right) \right)]$$

$$E\left[ XY \right]=E[\beta_{1}\beta_{2}Z^{2}+\beta_{1}ZN\left( 0,\sigma_{1} \right)+\beta_{2}ZN\left( 0,\sigma_{2} \right)+N\left( 0,\sigma_{2} \right)N\left( 0,\sigma_{1} \right)]$$

$$E\left[ XY \right]=E\left[ \beta_{1}\beta_{2}Z^{2} \right]+\beta_{1}E\left[ Z \right]E[N\left( 0,\sigma_{1} \right)+\beta_{2}E\left[ Z \right]E\left[ N\left( 0,\sigma_{2} \right) \right]+E\left[ N\left( 0,\sigma_{1} \right)*N\left( 0,\sigma_{2} \right) \right]$$

since the normal distributions are independent

$$E\left[ N\left( 0,\sigma_{1} \right)*N\left( 0,\sigma_{2} \right) \right]=0$$

the following relationship is obtained

$E\left[ XY \right]=\beta_{1}\beta_{2}E[Z^{2}]$ [5]

by substituting in equations 2-5 into equation 1

$$cov\left( X,Y \right)=\beta_{1}\beta_{2}E\left[ Z^{2} \right]-\beta_{1}\beta_{2}{E[Z]}^{2}$$

$$cov\left( X,Y \right)=\beta_{1}\beta_{2}(E\left[ Z^{2} \right]-{E[Z]}^{2}$$

$$cov\left( X,Y \right)=\beta_{1}\beta_{2}\sigma_{Z}^{2}$$

it can be shown for a linear relationship that the relationship between slope and Pearson correlations is given by

$$\beta_{i}=\rho_{iZ}\frac{\sigma_{i}}{\sigma_{Z}}$$

and thus

$$cov\left( X,Y \right)=\rho_{XZ}\rho_{YZ}\sigma_{X}\sigma_{Y}$$

$$\rho_{XY}=\rho_{XZ}\rho_{YZ}$$

as stated.

The histotype only models are fundamentally linear models of histotype average IC50’s. Not surprisingly, when we look at the product of the Pearson correlation between average histotype IC50’s in the training data and the values predicted by the models, $\rho_{HA}$, and then the correlation of the experimental values in the testing set with the average histotype IC50 in the training set $\rho_{EA}$, it is in close accordance with the observed correlation in the histotype models. As these values represent the maximum correlation that could be achieved by histotype alone it is clear that increased performance in such drugs as bleomycin, bortezomib, doxorubicin, etoposide, SN-38, and 5-flurouracil is a result of directly capturing cell based drug response (Sup figure 3 A and B). Additionally, there is a marked difference between the performance of the CTR2 models in NLSVR and PCR. The purpose of the CTR2 models was to eliminate any features whose variability could be directly attributed to drug response. Therefore, with respect to drug response, we would expect that the variability would be only due to histotype. Indeed, this is what we see in most PCR models, however, NLSVR CTR2 models perform substantially better. This suggests that while these genes have no direct relationship with drug response that, such as in vinblastine, SN-38 and 5-flurouracil (Sup figure 3 B), non-linear and linear combinations of these features are relevant to drug response. This is illustrative of another weakness in terms correlation based feature selection, it cannot capture features that result from interactions between genomic features. Therefore, any method of feature selection which focuses solely on the relationship between one genomic feature and drug response could potentially overlook features that result from complex interactions between multiple features.

**Elastic Net Models:**

Elastic nets one one of the best performing models in the NCI-DREAM challenge (1). Additionally, elastic nets come from a class of methods which can incorporate feature selection directly into model construction as opposed to filter feature selection which must be done prior to model fitting. In order to see if this could introduce any improvement we implemented elastic nets on all 15 drugs. As the other models the parameters were optimized using 10-fold nested cross-validation. The elastic nets had an average spearman correlation of 0.314 which performed better than ANN (0.1551) and SVRLN (0.2957) performed about similar to PCR (0.3139) and less than NLSVR (0.3264) (Supplementary fig 4). However, models for Cisplatin defaulted to a single value. In terms of WPC scores 0.576 which is comparable to CTR1 PCR models and CTR2 NLSVR models (Table 4 Main Text).

**Supplementary Figure 4.** Elastic net models compared to NLSVR, PCR, SVRLN, and ANN

**Validation Strategies:**

An important consideration in modeling is the strategy used to validate or access the performance of models. The simplest cross validation strategy divides the dataset into K folds

Then trains the model and K-1 folds and validates on the Kth fold this process is repeated for all K-folds giving an estimate of the average performance of the model as well as the variability in models due to sampling. However, when or complex task are added such as feature selection and hyperparameter optimization this can can yields over-optimistic results in what is called “data leakage” or “data snooping”. Filter based feature selection is performed prior to modeling but if this is done on the entirety data set the model has already seen aspects of the data and can create a bias. A more robust method is to leave out a portion of the dataset and then use the remaining portion of the dataset to do feature selection after which the hyperparameters can be optimized using a cross validation strategy where a parameter combination is used to train a model using a portion of the dataset validating on the remaining portion of the data and then repeating this several times to get an idea of the average performance the hyparameter values yield. This is then done for several other combinations of hyperparameters searching for the optimal values. This can then be repeated for several different samples of the data to get an idea of a general modeling approach performs. Sampling of the data can be done in several way, a leave one out (LOO) strategy, leaving a data point out for testing, a data point for validation, and use the remaining for training. Then data is then cycled until every data point has used in testing, training, and validating. LOO cross validation can be computationally intensive and the training error seen during testing might not generalize to a larger population. A K-folds strategy as described above is a more efficient and often preferred. A third method uses a monte-carlo approach a portion of the sample is chosen and the remaining data is used for training and validation, this can be done multiple times to get an idea of how a modeling approach performs. There key difference from a K-Fold approach and monte-carlo approach is that a K-folds approach ensures that very data point is used in testing, training, and validation where there is a chance in a monte-carlo approach a data point may never be used in testing or never used in training or validation. We choose to use a monte-carlo approach for a couple of reasons. Ultimately, the eventual objective is to use cell based models on tumor data which is very heterogeneous in nature and can be very genotype diverse. A monte-carlo method installs a stochastic component that might be more representative of a tumor environment. Secondly it allows for a greater number of splits maintaining percentages used in testing, training, and validation. For example, if I want to use 75 percent of data for training and validation and test on the remaining data a K-fold will only allow for 4 splits; furthermore, the proportion used in training and validation is comparable as with K-folds I am now only using ½ of the data to train where if I use monte-carlo I am using 0.56 of the total data. A stepwise description of our validation method is described below with included pseudo-code.

1. Break the data randomly into a (training/validation) set containing 75% of the data with the remaining 25 percent used for testing.
2. With only the training set apply a filter based feature selection approach (2) using spearman correlation to access the relevance of each gene to drug response.
3. Using the features, for every possible parameter combination, that meet a certain criterion, i.e. a p value less than a defined cutoff, break the training data again randomly into two sets of 75 percent for training and 25 percent for validation such that there is no overlap between the sets.
4. Train the model on the training set and access the performance on the validation set
5. Repeat 10 times
6. Select the parameter combination that yields that best average performance on all validation sets.
7. Retrain the data all of the training date using selected parameters.
8. Estimate the performance of the model using the independent test set.
9. Repeat steps 1-8 an additional 5 times.


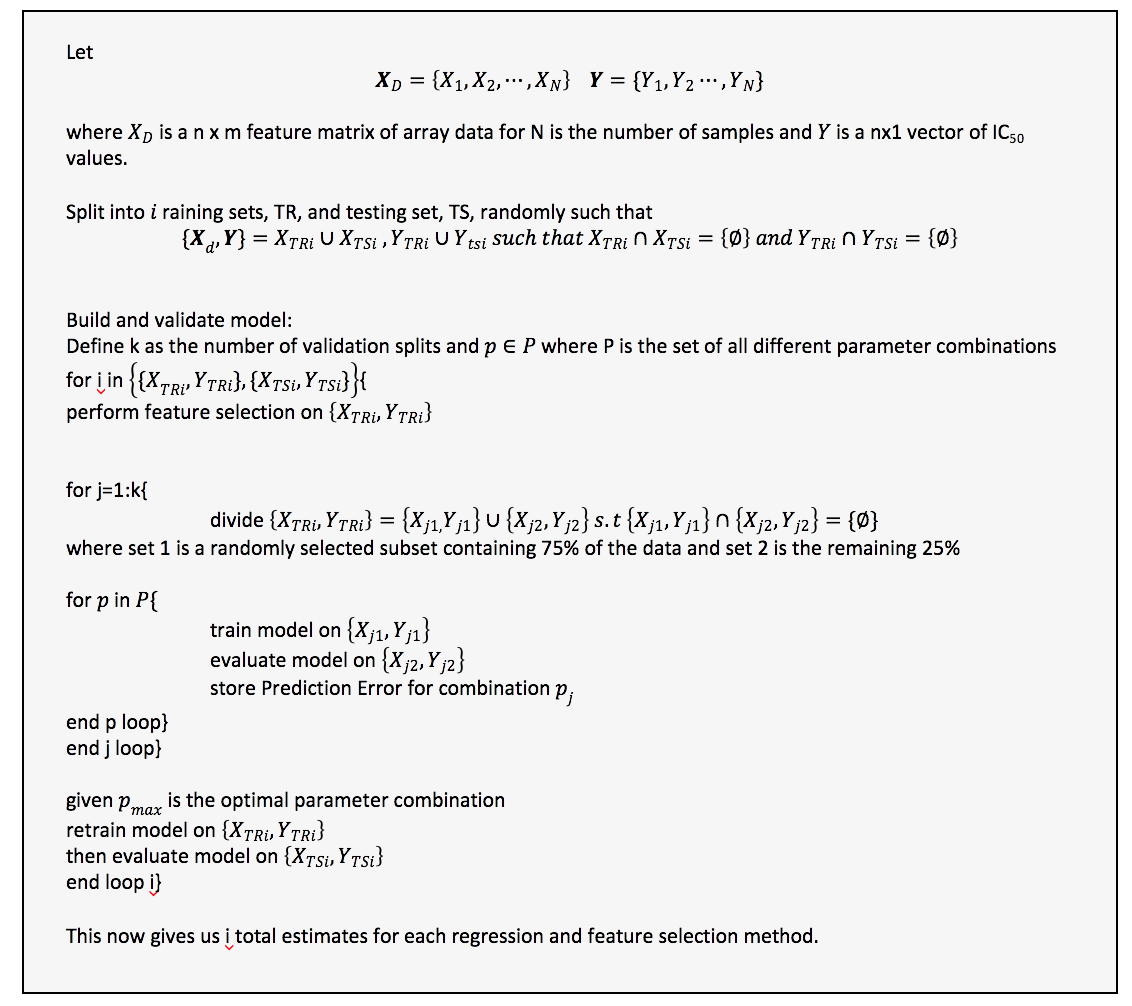


**Supplementary Figure 5:** Psuedo code outlined the testing, training, and validating processes.

Additionally, to verify that sampling strategies did not impact the major conclusions of our paper, the difference between performance between linear and non-linear methods is negligible, the influence of using correlation base feature selection has little influence is comparable to selecting a certain number of random features, and that histotype is a major driver of model performance. To do this we implemented a five-fold nested cross validation for 1000 random chosen DEGs, 1000 randomly chosen genes (CTR1), and 1000 randomly chosen genes that had no significant correlation with drug response (CTR2) for 14 of the 15 drugs (Cisplatin was excluded because of it randomly small sample size. These result were largely in agreement with previous models giving average correlations of (DEG: 0.337, CTR1: 0.296, CTR2: 0.307, Hist: 0.228, RCTR: -0.01) for nested cross validation and compared to (DEG: 0.32, CTR1: 0.312, CTR2: 0.294, Hist: 0.23, RCTR 0.01) for SVR in the original experiments. Likewise, in PCR (DEG: 0.311, CTR1: 0.243, CTR2: 0.253, Hist 0.2367, RCTR: 0.024) using nested cross validation (DEG: 0.303, CTR1: 0.282, CTR2: 0.235, Hist: 0.227, RCTR: -0.008) for the original models (Sfig 6).

Supplementary Figure 6: A. Average Spearman correlation values for 14 drugs where M1 is the cross validation strategy outlined in Sfig5 and M2 is the 5-fold nested cross validation for 1000 randomly chosen DEGs and one hot encoded (OHE) histotype models. B. Spearman correlation for OHE models (x-axis) vs 1000 Randomly chosen DEGs where M1 and M2 defined the same as in A.

**Histotype Distribution:**

The GDSC is composed of 1001 total cell lines and 55 histotypes, however the number of cells represented is highly variable ranging from 67 for Lung Non Small Cell Carcinoma to a single cell line for the adrenal gland averaging about 18.2 cell lines. Additionally, because we removed several cell lines which had a reported IC50 above the maximum concentration the largest dataset, mitomycin, carried 759 cells. So ultimately the number of cell lines could vary from drug to drug and since we used a monte-carlo method for sampling the histotype distribution could be very different between testing and training sets as well vary from set to set. Thus, a question arose as to whether the distribution of histotypes could effect the overall performance and additionally, would a more uniform distribution effect the influence of histotype on model performance. To answer this question we took a sample of ten random cell lines for each histotype on the ten most drugs with sufficient histotype diversity which resulted in 150 cell lines. Additionally, as a control 150 random cell lines were selected from the GDSC for each drug. Using 1000 random CBF selected features five fold nested cross validation was performed on both data sets as well as a histotype control for each different data set using non-linear support vector regression. Both the DEG models (stratified: 0.175, Random 0.176) and histotype models (stratified 0.1266, and 0.13). Additionally, the performance in both sets of DEG models correlated with the predictive strength of histotype models consistent with both GDSC and NCI60 (Sfig 7). This suggests that the ability of a model to associate variability in drug response and better performance is robust to stochastic variation in histotype distribution when sampled from a given population of cells.

Supplementary Figure 7: Spearman correlation for the OHE histotype models plotted against the Spearman correlation for DEG models for uniform where each histotype is represented by ten cells and random where cells are selected randomly from the GDSC.

**Additional Figures:**

1. Costello JC, Heiser LM, Georgii E, Gönen M, Menden MP, Wang NJ, et al. A community effort to assess and improve drug sensitivity prediction algorithms. Nature biotechnology. 2014;32(12):1202-12.
